# Problems with enuresis management—A personal view

**DOI:** 10.3389/fped.2022.1044302

**Published:** 2022-11-04

**Authors:** Tryggve Nevéus

**Affiliations:** Department of Women’s and Children’s Health, Uppsala University, Uppsala, Sweden

**Keywords:** management, voiding chart, daytime incontinence, constipation, enuresis alarm, desmopressin, urotherapy, enuresis

## Abstract

Much has happened since the end of the era when enuresis was blamed on the parents or the children themselves. Still, there are large gaps in our knowledge and large parts of modern enuresis management guidelines are (still) not based on firm evidence. In this review I will question the following commonly made assumptions regarding enuresis evaluation and treatment:

• It is important to subdivide enuresis according to the presence of daytime symptoms
• Voiding charts are crucial in the primary evaluation of the enuretic child
• All children with enuresis need to be screened for behavioral or psychiatric issues
• Concomittant daytime incontinence needs to be successfully treated before addressing the enuresis
• Concomittant constipation needs to be successfully treated before addressing the enuresis
• Urotherapy is a first-line treatment against enuresis

In this review I will argue that much of what we do with these children is based more on experience and well-meant but poorly supported assumptions than on evidence. Some advice and therapies are probably ineffective whereas for other treatments we lack reliable predictors of treatment response. More research is obviously needed, but awaiting new results enuresis management could be substantially simplified.

## Introduction

Enuresis used to be viewed as a purely psychiatric disorder. Until the 1980s the evaluation of bedwetting children was focused on behavior, early trauma and other psychological factors, and therapy—if any therapy was advocated—was usually psychotherapy in various forms.

But since the seminal work in the late 80s by the Aarhus group we know more (1). Enuresis is familial in the majority of cases and caused by various combinations of nocturnal polyuria, nocturnal detrusor overactivity and high arousal thresholds (2). And the link between enuresis and psychiatric/psychological issues is due to on the one hand poor self esteem, casued by the wetting (3), and on the other an overrepresentation of children with neuropsychiatric disorders such as attention-deficit/hyperactivity disorder (ADHD) (4).

The recommended strategy for managing these children has changed accordingly, as reflected by international guidelines (5–10). Now, the evaluation of the enuretic child is heavily focused on the bladder and on urine production. The children are instructed to complete voiding charts, recording daytime voiding frequency and voided volumes as well as nocturnal urine production (10). Based on the anamnesis and the voiding charts the children are subdivided into monosymptomatic and nonmonosymptomatic groups, the latter having concomittant daytime lower urinary tract (LUT) symptoms and/or a daytime micturition frequency that is regarded as abnormal (11). Signs of constipation are also actively sought for (7), using the Rome IV criteria (12). The need to screen all enuretic children for behavioral issues and signs of neuropsychiatric disorders is also underlined (4, 13).

Nowadays, children with enuresis are expected to be taken seriously and the wait-and-see attitude is no longer accepted, at least for children aged six years or more. Neither is psychotherapy advocated as a primary (or indeed secondary) therapy. Instead the recommendation is often given that the LUT function of these children be “normalised” by the institution of regular drinking (10) and voiding habits and correct voiding posture: i.e., basic urotherapy (5, 6). This is recommended to be the first-line therapy at least for children with NMNE (5, 7). Likewise, treatment of concomittant constipation is recommended (9). For other enuretic children, and for those still wet after urotherapy, either desmopressin or the enuresis alarm are recommended. Anticholinergics and tricyclic antidepressants are recommended as second- and third-line therapies, respectively (5).

The new prevailing strategy for the management of children with enuresis is surely a great step forward compared with the views of several decades ago, but there are still problems. Much of what we now do is (still) based not on firm evidence but on experience and assumptions. These assumptions are not by any means unreasonable, just not properly tested.

The aim of this review is to scrutinize some of the central assumptions underlying modern enuresis management. By doing this I do not mean neither to polemize or criticize the experts nor distance myself from my contribution to the current guideline documents but, hopefully, to underline fields needing more research and to suggest ways that—pending that research—enuresis management may be simplified, at least outside the university setting.

## 1st assumption: it is important to subdivide enuresis according to the presence of daytime symptoms

According to the International Children's Continence Society (ICCS) nocturnal enuresis can, and should, be subdivided into monosymptomatic nocturnal enuresis (MNE) and nonmonosymptomatic nocturnal enuresis (NMNE) on the basis of whether daytime symptoms of LUT dysfunction are also present or not (11, 14). These daytime symptoms include daytime incontinence and urgency but also somewhat arbitrarily defined high or low micturition frequency—i.e., more than seven or less than four voidings per day. Consequently, no child can be assigned to the MNE group without completing a voiding chart.

The MNE/NMNE definitions were an update of the previous terminology document that stated that MNE, defined only as enuresis without daytime incontinence, involved “urodynamically normal voidings” whereas NMNE did not (15). When we updated this terminology we recognized that there are other relevant daytime symptoms, not just incontinence, and we were not so sure that the enuretic incident of the child with MNE were really urodynamically normal.

Still, we did make assumptions regarding the underlying pathogenesis and the expected response to therapy. The argument went something like this: (1) we know that detrusor overactivity is one crucial pathogenic factor behind enuresis, (2) we assume that symptoms such as daytime incontinence and urgency, and findings such as a high daytime micturition frequency, indicate underlying detrusor overactivity (16, 17), (3) we believe that children with enuresis due to detrusor overactivity should be treated differently than those without this condition. Among first-line therapies, desmopressin was assumed to be a poor choice to children with NMNE whereas urotherapy was assumed to help these children (5).

We have usually assumed that children with NMNE constitute the minority, but this has been questioned (18–23, 29). If voiding charts are included in the workup and even subtle urgency symptoms are taken into account, perhaps NMNE is the condition of the majority. The more you ask, the more you will find. Which child doesn't sometimes have to rush to the toilet?

Urgency is a particularly problematic symptom. It is assumed to indicate underlying detrusor overactivity (16), a phenomenon that can only be diagnosed by invasive cystometry. Both among adults and children urgency alone is sufficient for the patient to be assigned the diagnosis overactive bladder (11, 24). And this symptom alone suffices for the child's bedwetting to be called nonmonosymptomatic, and thus require specific therapy or even referral to a specialist (8). We may ask the child something like this: “when you need to pee, do you have to rush or can you wait a little while?”. But if the child answers yes, what does this really mean? That we are dealing with uninhibited detrusor contractions (i.e., detrusor overactivity) or that sometimes that child is so absorbed with activities that it doesn't notice the need to void until it's nearly too late? Cystometric studies in adults show a disturbingly poor correlation between self-reported urgency and actual detrusor overactivity (25–28). We shouldn't expect the correlation to be better among children.

Looking for studies addressing whether children with MNE and NMNE require different therapies the result is meager. In very many studies, perhaps the majority, only children with presumed MNE have been included. And even though many of those studies have not had a verified “high” or “low” (daytime) micturition frequency among the exclusion criteria (and consequently probably a proportion of children with actual NMNE have been included) the result is that we know very little about which therapy works and does not work in children with NMNE. Studies expressly including children with both MNE and NMNE while clearly characterizing them, and giving them the same therapy while looking for differences in therapy response between the groups, are very few indeed (29). The response to the alarm at least doesn't seem to differ between the groups (30), whereas desmopressin response is slightly less favorable among children with NMNE (31).

Perhaps the only remaining argument for giving different treatment across the MNE/NMNE divide is that if there is nocturnal polyuria there is no need to assume concomittant detrusor overactivity and desmopressin could be tested straight away. But even this assumption has not been properly tested, due to a lack of studies of desmopressin treatment in children with properly defined NMNE (31). And how do we start the treatment of the child with both nocturnal polyuria and daytime urgency?

In the latest enuresis guideline document from the ICCS these uncertainties have been acknowledged by joining the previously separate NMNE and MNE documents (6, 7) into one (5). But we still recommend partly different therapies for the two subgroups.

In future guidelines, I suggest that much less emphasis is put on the MNE-NMNE subdivision.

## 2nd assumption: voiding charts are crucial in the primary evaluation of the enuretic child

Voiding charts are usually promoted as a highly recommended component of the evaluation of children with enuresis (5, 9). The standard voiding chart includes the measurement of (daytime) voided volumes and frequency during 2–3 days as well as the measurement of nocturnal urine production *via* weighing of diapers or sheet covers (11).

The basis for mandating voiding charts is partly the same as that for differentiating between MNE and NMNE described above, i.e., by looking for signs of underlying detrusor overactivity we assume that we are helped in our choice of first-line therapy. And as mentioned above, if we suspect detrusor overactivity we assume that urotherapy will and desmopressin will not work. We also suspect that anticholinergics may be succesful as a second-line therapy. Furthermore, by detecting nocturnal polyuria we assume that desmopressin therapy will be successful.

Leaving the nocturnal urine production aspect aside for a moment we must ask ourselves: what do we really measure when we document the child's micturitions during a few days? Bladder function or behavior? How many of the voidings happen because (a) the parents told the child to void, (b) the child believed that it was supposed to void, (c) it was socially convenient to go to the toilet, (d) the bladder was full, or (e) uninhibited detrusor contractions? It's really only the last reason for voiding which is interesting for us, the others are just obscuring the picture.

Sadly, the evidence for a link between voiding chart data such as voided volumes or voiding frequency and cystometric findings is very tenuous (25, 32). And the fact that daytime voided volumes do not clearly correlate to nocturnal voided volume, i.e., the enuresis volume, indicate that the link between daytime and nocturnal bladder function is not straightforward (33, 34). Likewise, it is known that children may have stable bladders during daytime and nocturnal detrusor overactivity (35). Furthermore, the voiding frequency almost by definition gives only limited information, since this variable depends just as much on fluid intake as on bladder function. In short: the voiding chart is probably much too blunt an instrument for us to make conclusions regarding nocturnal detrusor overactivity.

The value of the voiding chart as a predictor of enuresis therapy response is also meager. There are data suggesting that normal voided volumes are more common among desmopressin responders (36–38), but these data are not unequivocal, and the predictive value regarding the alarm is probably negligible (39–41).

It should be added here that in the treatment of daytime incontinence, as opposed to enuresis, the voiding chart can be motivated as part of *therapy*, regardless of whether it is useful as a diagnostic tool or not. By documenting the micturitions in a chart it is probably easier to adhere to the voiding schedule according to the instructions given (42).

Regarding nocturnal urine production the situation is different. It has been shown that nocturnal polyuria (i.e., a nocturnal urine production in excess of 130% of the expected bladder capacity for the child's age) predicts a likelihood that desmopressin will work (43). However, there are conflicting studies (44), and the correlation between nocturnal polyuria and desmopressin response is not perfect (45, 46). The cut-off at 130% is also quite arbitrarily chosen (11).

A problem here is that nocturnal urine production measurements, to be reasonably reliable as predictors of desmopressin response, need to be performed several times. Once or twice is not enough (46, 47). And the measurements require much commitment and motivation from the families. Many children risk being lost to follow-up—and left untreated—if we demand that they deliver measurements of nocturnal urine production before moving on with therapy. Furthermore, it is probably utopistic to demand that these measurements be performed under the guidance of general practitioners or school nurses. Thus I suspect that this strategy is difficult to implicate as a truly first-line investigation in primary care. In that setting it is probably much easier just to test if desmopressin works.

It could also be questioned—given the less than perfect correlation between nocturnal urine production and desmopressin response—whether the absence of nocturnal polyuria in a child who does not respond to the enuresis alarm means that we should let anticholinergics or tricyclics be the next step before testing desmopressin. I think not.

## 3rd assumption: all children with enuresis need to be screened for behavioural or psychiatric issues

Although old psychodynamic explanations regarding enuresis pathogenesis are clearly obsolete it has been convincingly shown that children with enuresis—especially if they also have daytime incontinence and/or fecal incontinence—are more prone to behavioral problems or neuropsychiatric disorders than their nonenuretic peers (13, 48, 49). This is taken as grounds for recommending that all children with enuresis be at least screened for such issues already at the initial evaluation (4).

The central motivation for this recommendation is neither that we consider the psychiatric issues to be causative nor that treatment of them will by itself make the children dry. Instead it rests on the assumption that concomittant problems such as ADHD will negatively influence treatment response.

This assumption does not seem unreasonable regarding the enuresis alarm or urotherapy—treatments that demand much active cooperation from the child. But there is no reason to believe that response to pharmacological treatment is affected (50).

And although it seems fair to suspect that successful alarm treatment is difficult to achieve in a child who scores positive on a screening tool for, say, ADHD, this has not been put to the test in prospective studies. Intriguingly, in an American study comparing 95 enuretic children with ADHD and 95 children without ADHD no differences were found regarding alarm treatment results (51). Maybe the children who cannot adhere to therapy due to psychiatric issues are those children who would need psychiatric therapy anyway, regardless of their enuresis. We do not, yet, have enough scientific support to conclude that children with enuresis who score positive on screening instruments for psychiatric conditions but do not have substantial problems with school or other social interactions should be referred to a child psychologist or psychiatrist. More research is needed before we can draw such a conclusion.

It should be kept in mind that in many countries and settings child psychiatry and psychology are scarce resources. Can the healthcare system take care of all the new referrals, should this recommendation be followed? Thus, given the current state of evidence, I would suggest that if screening tools are used and indicate behavioral/psychiatric issues, then the child should not be automatically referred unless there are also substantial problems with social interaction apart from the wetting. Or perhaps wait until one serious alarm attempt has been tried and failed.

## 4th assumption: concomittant daytime incontinence, if present, needs to be successfully treated before addressing the enuresis

This recommendation is also based on assumptions regarding detrusor overactivity, which is the main cause behind daytime incontinence (52). First, by successfully treating daytime incontinence, with urotherapy, there is a fair chance that the bedwetting will disappear as well. Or so it is presumed. Second, perhaps succesful enuresis therapy is impossible if the daytime problem is not first addressed. A third argument is that the daytime incontinence is a greater problem for the child, with a higher risk for public embarrasment, and that it should be treated first for that reason.

But we have no firm evidence for the truth of either the first or the second of these arguments. In fact, there are earlier studies indicating that it may be the other way around, i.e., that enuresis alarm treatment may work regardless of concomittant daytime incontinence (53), that it may actually improve the daytime situation (53) and that treatment of daytime incontinence with urotherapy has no effect against concomittant enuresis (54). There is a disturbing lack of recent studies addressing these assumptions.

The third argument, about the impact of daytime incontinence being greater for the child, may certainly be true but that's not for us to decide.

Thus, pending new evidence, the best strategy is probably to let the families decide about which problem to address first. Or treat both conditions simultaneously. As the evidence now stands we have no grounds for delaying the enuresis therapy just because the child also wets during daytime.

## 5th assumption: concomittant constipation needs to be successfully treated before addressing the enuresis

This assumption is based on the well-supported link between bowel and LUT function, especially the connection between constipation and detrusor overactivity (55, 56). The bladder and bowel may influence each other on all levels from the pelvis to the cerebral cortex (57–59). Constipation is common among children with daytime incontinence or recurrent urinary tract infections and *vice versa* (60), and bowel management can alleviate—and is, indeed, mandatory—in these conditions (61, 62).

In analogy to treatment of daytime incontinence it is assumed that treatment of concomittant constipation will either make the enuretic child dry or be a necessary prerequisite for treatment directed at the enuresis to be effective.

But the link between enuresis and constipation is not as clear-cut as that between daytime incontinence and constipation. There are some recent studies that show an epidemiological overrepresentation (63, 64) and some that don't (65–68). Probably, if the enuresis is monosymptomatic, constipation is not more common than for other children (69, 70). And the evidence for an antienuretic effect of treatment of constipation is much more meager (61). There are no prospective, controlled studies on the effect of laxative treatment on enuresis.

For those with stomach pains or fecal incontinence this is no problem, they need to be treated for their constipation regardless of the wetting. But except for one recent study at our centre there are no studies on the value of constipation treatment for enuretic children who are not much bothered by bowel-related symptoms. We found that enuretic children with constipation according to the Rome IV criteria and/or rectal distension had no benefit of enemas and laxative therapy, at least not during the first month (71).

It should be remembered that constipation therapy is quite labor-intensive, including frequent rectal enemas or high-dose debulking agents during the first weeks followed by several months of maintenance therapy. Just providing polyethylene glycol and some good advice will not be enough (72). Thus, the motivation to stick to the therapy when the child has no subjective bowel-related problems, will be a problem.

Thus, awaiting evidence of a clear antienuretic benefit of constipation therapy we should perhaps reserve those efforts to enuretic children with stomach-related complaints, and/or concomittant daytime incontinence and those not responding to first-line therapy, not just those that fulfill the Rome criteria.

## 6th assumption: urotherapy is a first-line treatment against enuresis

The following set of advice—which fits well within the ICCS definition of basic urotherapy (73)—has regularly been recommended as a first-line treatment of children with enuresis:

• Demystification, explanation and removal of guilt
• Regular voidings according to a schedule
• Regular drinking habits
• Correct voiding position with support for the thighs

The first item on the above list is, of course, warranted regardless of therapeutic efficacy. This is part of good doctoring/nursing and will not be questioned.

The motivations behind these recommendations are several. One is the shared underlying detrusor overactivity in both enuresis and daytime incontinence, and the fact that urotherapy is the established firstline therapy of the latter condition (42). It is not far-fetched to assume that by normalizing bladder function during the day beneficial effects will also be experienced during the night.

Another motivation is probably clinical experience: many nurses have met children who gradually get drier at night as the months pass and they work with the voiding schedules. But this is anecdotal evidence and clinical impressions can be deceptive. Many of those children would perhaps had become dry anyway, and what about the families that didn't show up for the next appointment?

But the recommendation does not rest on evidence. The studies on urotherapy in enuresis have all been uncontrolled and most have either been retrospective and/or failed to take the high drop-out rate into account (54, 74–77). On the other hand, the first randomized, controlled evaluation showed no effect at all of four weeks of urotherapy (78). And our recent randomized, controlled trial comparing eight weeks of urotherapy with the enuresis alarm and no therapy showed that only the alarm provided any benefit; urotherapy was just as good as no therapy (79). It may be argued that treatment during a longer time period would perhaps show a better result, but the workload and time demanded by such a treatment would certainly disqualify its use as a first-line therapy.

Furthermore, although urotherapy certainly isn't harmful, and the alliance formed between the therapist and the child (provided the family returns for the next appointment) may boost the child's self esteem, it should be noted that it is time-consuming for both the family and the healthcare provider.

Based on these considerations it is fair to say that daytime urotherapy has no place in the initial treatment of children with enuresis.

## Discussion and recommendations

The purpose of this review has not been to criticize the experts behind the existing guidelines. I have myself been very active in the creation of the relevant ICCS documents a contribution which I do not regret. These documents have been based on the available evidence, and, whenever evidence has been unavailable, the collective clinical experiences and reasonable assumptions of the experts. My aim here has been to highlight the many existing uncertainties and the areas in which recent evidence contradict our previous assumptions. My views and recommendations are summarized in Table 1 below.

**Table 1:** Problems with current recommendations and suggested further research and guideline changes.

Recommendations	Problems	Suggested research	Suggested interim guideline changes
All enuretic children need to be differentiated into monosymptomatic and nonmonosymptomatic subgroups before choosing therapy	Weak and contradictory evidence. Most studies have not subdivided according to guidelines	Prospective studies, examining the efficacy of first-line enuresis therapy on representative samples of unselected enuretic children. The predictive value (if any) of daytime voiding chart data and daytime bladder habits is assessed.	During initial evaluation in primary care put less emphasis on daytime voiding habits and voiding chart data and more on warning signs. If no warning signs, proceed directly to treatment with the alarm or desmopressin
All enuretic children need to complete voiding charts before choosing therapy	Firm evidence only for nocturnal polyuria predicting desmopressin response. Value of daytime bladder data questionable. Difficult to implicate in primary care.		
All enuretic children need screening for psychiatric issues at initial evaluation	Insufficient evidence if this will influence final outcome. Socio-economic issues	Prospective studies of enuretic children with and without psychiatric comorbidity given the same antienuric treatment.	Evaluate psychiatric issues only if the child has substantial problems with social interaction
Concomittant daytime incontinence needs to be treated before adressing the enuresis	Insufficient and contradictory evidence	Prospective studies on children with combined enuresis and daytime incontinence randomized to urotherapy or no urotherapy before antienuretic therapy	Don’t let daytime incontinence delay treatment of the enuresis
Concomittant constipation needs to be treated before adressing the enuresis	Insufficient and contradictory evidence	Prospective studies on children with enuresis and constipation randomized to laxatives or no laxatives before antienuretic therapy	Treat constipation if the child has stomach ache, fecal incontinence or daytime urinary incontinence, or if the enuresis is therapy-resistant
Urotherapy is a first-line therapy against enuresis, at least for nonmonosymptomatic children	No supporting evidence. Recent studies show no effect	(Randomized studies of urotherapy in therapy-resistant enuresis)	Don’t use urotherapy as a first-line therapy in enuresis

It should be obvious that there are many areas in which new research is sorely needed, and these include such basic questions as what to focus on during the primary evaluation and how to choose first-line therapy for vast numbers of children. Most of the research needed is not hi-tech or expensive, but the impact for the many affected children will be considerable. Here are some suggested fields that deserve further study:

• The role (if any) of voiding charts in the evaluation of enuretic children
• Desmopressin and alarm response in enuretic children with concomittant daytime symptoms
• The effect of treatment for neuropsychiatric disorders on response to first-line enuresis therapy
• Studies on the need for, or benefit of, treatment of non-bothersome constipation in children with enuresis

Based on the available evidence today, I suggest that the following changes are made to the recommended enuresis management:

• Put less emphasis on the differentiation of enuresis into monosymptomatic and nonmonosymptomatic varieties
• If voiding charts are used, make sure that families who don’t manage to complete them are not lost
• Don't let concomittant daytime incontinence be a contraindication to enuresis treatment
• Stop using (daytime) urotherapy as a treatment of enuresis

Situations with strained resources, for the families and/or the healthcare system, deserve special mention. Here, the perfect can be the enemy of the good. We cannot expect that all families of enuretic children who seek healthcare assistance for the first time are able to adhere to time-consuming or labor-intensive evaluation methods or therapies. Likewise, we cannot expect primary care healthcare professionals without expertise regarding the pediatric LUT to be able to conduct state-of-the art enuresis management the way we experts would do it. In this setting—awaiting new research findings—I suggest the following cornerstones of a simplified, “bare-bones” enuresis management strategy for primary care:

• At the first visit, focus on warning signals that indicate serious underlying conditions (general symptoms or weight loss, excessive thirst with a need to drink at night, poor urinary stream with a need to strain to void)
• No need for voiding charts or measurement of nocturnal urine production
• Do not let concomittant daytime incontinence delay enuresis therapy
• Treat constipation only if it bothers the child or if there is also daytime incontinence
• Start directly with alarm or desmopressin treatment according to family preferences
• Seek the help of a psychiatrist/psychologist if the child has substantial problems with social interaction or school, but do not let this delay enuresis therapy

This way, an immense benefit could be gained for millions of children while we keep doing research in order to make future management strategies more evidence-based.

## References

[1] Rittig S Knudsen UB Nørgaard JP Pedersen EB Djurhuus JC. Abnormal diurnal rhythm of plasma Vasopressin and urinary output in patients with enuresis. Am J Physiol. (1989) 256:F664–71. 10.1152/ajprenal.1989.256.4.F664 2705537

[2] Nevéus T. The pathogenesis of enuresis—towards a new understanding. Int J Urol. (2017) 24(3):174–82. 10.1111/iju.13310 28208214

[3] Hägglöf B Andrén O Bergström E Marklund L Wendelius M. Self-esteem before and after treatment in children with nocturnal enuresis and urinary incontinence. Scand J Urol Nephrol. (1997) 31(Suppl 183):79–82. PMID: 9165615

[4] von Gontard A Baeyens D Van Hoecke E Warzak WJ Bachmann C. Psychological and psychiatric issues in urinary and fecal incontinence. J Urol. (2011) 185(6):2303–7. 10.1016/j.juro.2011.02.040 21349549

[5] Nevéus T Fonseca E Franco I Kawauchi A Kovacevic L Nieuwhof-Leppink AJ Management and treatment of nocturnal enuresis—an updated standardization document from the International Children’s Continence Society. J Ped Urol. (2020) 16:10–9. 10.1016/j.jpurol.2019.12.020 32278657

[6] Nevéus T Eggert P Evans J Macedo A Rittig S Tekgül S Evaluation and treatment of monosymptomatic enuresis—a standardisation document from the International Children’s Continence Society (ICCS). J Urol. (2010) 183:441–7. 10.1016/j.juro.2009.10.043 20006865

[7] Franco I von Gontard A De Gennaro M. Evaluation and treatment of nonmonosymptomatic nocturnal enuresis: a standardization document from the International Children’s Continence Society. J Pediatr Urol. (2012) 9(2):234–43. 10.1016/j.jpurol.2012.10.026 23260268

[8] Vande Walle J Rittig S Bauer S Eggert P Marschall-Kehrel D Tekgül S. Practical consensus guidelines for the management of enuresis. Eur J Pediatr. (2012) 171:971–83. 10.1007/s00431-012-1687-7 22362256 PMC3357467

[9] Vande Walle J Rittig S Tekgül S Austin P Yang SS Lopez PJ Enuresis: practical guidelines for primary care. Br J Gen Pract. (2017) 67(660):328–9. 10.3399/bjgp17X691337 28533201 PMC5565868

[10] Bogaert G Stein R Undre S Nijman RJM Quadackers J ‘t Hoen L Practical recommendations of the EAU-ESPU guidelines committee for monosymptomatic enuresis-bedwetting. Neurourol Urodyn. (2020) 39:489–97. 10.1002/nau.24239 31793066

[11] Austin P Bauer S Bower W Chase J Franco I Hoebeke P The standardization of terminology of lower urinary tract function in children and adolescents: update report from the standardization committee of the International Children’s Continence Society. J Urol. (2014) 191(6):1863–5. 10.1016/j.juro.2014.01.110 24508614

[12] Benninga MA Faure C Hyman PE St James Roberts I Schechter NL Nurko S. Childhood functional gastrointestinal disorders: neonate/toddler. Gastroenterology. (2016) 150(6):1443–55.e2. 10.1053/j.gastro.2016.02.016 27144631

[13] von Gontard A Equit M. Comorbidity of ADHD and incontinence in children. Eur J Child Adolesc Psychiatry. (2015) 24(2):127–40. 10.1007/s00787-014-0577-0 24980793

[14] Nevéus T von Gontard A Hoebeke P Hjälmås K Bauer S Bower W The standardization of terminology of lower urinary tract function in children and adolescents: report from the standardisation committee of the International Children’s Continence Society (ICCS). J Urol. (2006) 176(1):314–24. 10.1016/S0022-5347(06)00305-3 16753432

[15] Nørgaard JP van Gool JD Hjälmås K Djurhuus JC Hellström A-L. Standardization and definitions in lower urinary tract dysfunction in children. Br J Urol. (1998) 81(Suppl 3):1–16. 10.1046/j.1464-410x.1998.00025.x 9634012

[16] Bauer SB Retik AB Colodny AH Hallett M Khoshbin S Dyro FM. The unstable bladder of childhood. Urol Clin North Am. (1980) 7:321–36. 10.1016/S0094-0143(21)01235-0 7404871

[17] Charalampous S Printza N Hashim H Bantouraki M Rompis V Ioannidis E Bladder wall thickness and urodynamic correlation in children with primary nocturnal enuresis. J Pediatr Urol. (2013) 9(3):334–8. 10.1016/j.jpurol.2012.04.008 22652388

[18] Fonseca EG Bordallo AP Garcia PK Munhoz C Silva CP. Lower urinary tract symptoms in enuretic and nonenuretic children. J Urol. (2009) 182:1978–83. 10.1016/j.juro.2009.04.083 19695589

[19] Chandra M Saharia R Hill V Shi Q. Prevalence of diurnal voiding symptoms and difficult arousal from sleep in children with nocturnal enuresis. J Urol. (2004) 172(1):311–6. 10.1097/01.ju.0000132363.36007.49 15201802

[20] Karamaria S Ranguelov N Hansen P De Boe V Verleyen P Segers N Impact of new vs. old International Children’s Continence Society standardization on the classification of treatment naïve enuresis children at screening: the value of voiding diaries and questionnaires. Front Pediatr. (2022) 10:862248. 10.3389/fped.2022.862248 35419322 PMC8995850

[21] Baek M Park KH Lee HE Kang JH Suh HJ Kim JH A nationwide epidemiological study of nocturnal enuresis in Korean adolescents and adults: population based cross sectional study. J Korean Med Sci. (2013) 28:1065–70. 10.3346/jkms.2013.28.7.1065 23853491 PMC3708079

[22] Bakker E van Sprundel M van der Auwera JC van Gool JD Wyndaele JJ. Voiding habits and wetting in a population of 4,332 Belgian schoolchildren aged between 10 and 14 years. Scand J Urol Nephrol. (2002) 36(5):354–62. 10.1080/003655902320783863 12487740

[23] Bower WF Moore KH Shepherd RB Adams RD. The epidemiology of childhood enuresis in Australia. Br J Urol. (1996) 78(4):602–6. 10.1046/j.1464-410X.1996.13618.x 8944518

[24] Abrams P Cardozo L Fall M Griffiths D Rosier P Ulmsten U The standardisation of terminology in lower urinary tract function. Neurourol Urodyn. (2002) 21:167–78. 10.1002/nau.10052 11857671

[25] Daan NM Schweitzer KJ van der Vaart CH. Associations between subjective overactive bladder symptoms and objective parameters on bladder diary and filling cystometry. Int Urogynecol J. (2012) 23(11):1619–24. 10.1007/s00192-012-1774-3 22543547 PMC3592260

[26] Ptashnyk T Hatzinger M Zeller FL Kirschner-Hermanns R. Overactive bladder syndrome—focus onto detrusor overactivity. Scand J Urol. (2021) 55:56–60. 10.1080/21681805.2020.1839130 33118417

[27] Michel MC Chapple CR. Basic mechanisms of urgency: preclinical and clinical evidence. Eur Urol. (2009) 56:298–308. 10.1016/j.eururo.2009.05.028 19523751

[28] Karaman MI Esen T Koçak T Akinci M Tellaloglu S. Rationale of urodynamic assessment in adult enuresis. Eur Urol. (1992) 21(2):138–40. 10.1159/000474820 1499614

[29] Kang BJ Chung JM Lee SD. Evaluation of functional bladder capacity in children with nocturnal enuresis according to type and treatment outcome. Res Rep Urol. (2020) 12:383–9. 10.2147/RRU.S267417 32984086 PMC7501990

[30] Kwak KW Park KH Baek M. The efficacy of enuresis alarm treatment in pharmacotherapy-resistant nocturnal enuresis. Urology. (2011) 77(1):200–4. 10.1016/j.urology.2010.06.050 20947143

[31] Rittig N Hagstroem S Mahler B Kamperis K Siggaard C Mikkelsen MM Outcome of a standardized approach to childhood urinary symptoms—long-term follow-up of 720 patients. Neurourol Urodyn. (2014) 33(5):475–81. 10.1002/nau.22447 23765698

[32] Guralnick ML Grimsby G Liss M Szabo A O’Connor C. Objective differences between overactive bladder patients with and without urodynamically proven detrusor overactivity. Int Urogynecol J. (2010) 21:325–9. 10.1007/s00192-009-1030-7 19907912

[33] Nevéus T. The amount of urine voided in bed by children with enuresis. J Ped Urol. (2019) 15:31.e1–5. 10.1016/j.jpurol.2018.08.006 30181098

[34] Kim JM Park JW Lee CS. Evaluation of nocturnal bladder capacity and nocturnal urine volume in nocturnal enuresis. J Pediatr Urol. (2014) 10(3):559–63. 10.1016/j.jpurol.2013.11.020 24388899

[35] Yeung CK Sit FK To LK Chiu HN Sihoe JD Lee E Reduction in nocturnal functional bladder capacity is a common factor in the pathogenesis of refractory nocturnal enuresis. BJU Int. (2002) 90(3):302–7. 10.1046/j.1464-410X.2002.02884.x 12133069

[36] Eller DA Austin PF Tanguay S Homsy YL. Daytime functional bladder capacity as a predictor of response to desmopressin in monosymptomatic nocturnal enuresis. Eur Urol. (1998) 33(Suppl 3):25–9. 10.1159/000052238 9599733

[37] Nevéus T. Oxybutynin, desmopressin and enuresis. J Urol. (2001) 166(6):2459–62. 10.1016/S0022-5347(05)65616-9 11696812

[38] Rushton HG Belman AB Zaontz MR Skoog SJ Sihelnik S. The influence of small functional bladder capacity and other predictors on the response to desmopressin in the management of monosymptomatic nocturnal enuresis. J Urol. (1996) 156:651–5. 10.1016/S0022-5347(01)65775-6 8683752

[39] Oredsson AF Jørgensen TM. Changes in nocturnal bladder capacity during treatment with the bell and pad for monosymptomatic nocturnal enuresis. J Urol. (1998) 160(1):166–9. 10.1016/S0022-5347(01)63082-9 9628642

[40] Berg IB Forsythe WI McGuire R. Response of bed wetting to the enuresis alarm: influence of psychiatric disturbance and maximum functional bladder capacity. Arch Dis Child. (1982) 57:394–6. 10.1136/adc.57.5.394 7092298 PMC1627550

[41] Hansen AF Jørgensen TM. Alarm treatment: influence on functional bladder capacity. Scand J Urol Nephrol. (1997) 31(Suppl 183):59–60. PMID: 9165610

[42] Chang SJ Van Laecke E Bauer SB von Gontard A Bägli D Bower WF Treatment of daytime urinary incontinence: a standardization document from the International Children’s Continence Society. Neurourol Urodyn. (2015) 36(1):43–50. 10.1002/nau.22911 26473630

[43] Hunsballe JM Hansen TK Rittig S Nørgaard JP Pedersen EB Djurhuus JC. Polyuric and non-polyuric bedwetting—pathogenetic differences in nocturnal enuresis. Scand J Urol Nephrol. (1995) S173:77–9. PMID: 8719573

[44] Akagawa S Tsuji S Akagawa Y Yamanouchi S Kimata T Kaneko K. Desmopressin response in nocturnal enuresis without nocturnal polyuria in Japanese children. Int J Urol. (2021) 28(9):964–8. 10.1111/iju.14615 34169597

[45] Kamperis K Rittig S Radvanska E Jørgensen KA Djurhuus JC. The effect of desmopressin on renal water and solute handling in desmopressin resistant monosymptomatic nocturnal enuresis. J Urol. (2008) 180(2):707–14. 10.1016/j.juro.2008.04.047 18554642

[46] Marzuillo P Marotta R Guarino S Fedele MC Palladino F Capalbo D “Frequently recurring” nocturnal polyuria is predictive of response to desmopressin in monosymptomatic nocturnal enuresis in childhood. J Pediatr Urol. (2019) 15(2):166.e1–7. 10.1016/j.jpurol.2018.11.004 30528650

[47] Hansen MN Rittig S Siggaard C Kamperis K Hvistendahl G Schaumburg HL Intra-individual variability in nighttime urine production and functional bladder capacity estimated by home recordings in patients with nocturnal enuresis. J Urol. (2001) 166(6):2452–5. 10.1016/S0022-5347(05)65614-5 11696810

[48] Kanata S Koike S Ando S Nishida A Usami S Yamasaki S Enuresis and hyperactivity-inattention in early adolescence: findings from a population-based survey in Tokyo (Tokyo Early Adolescence Survey). PLoS One. (2016) 11(7):e0158786. 10.1371/journal.pone.0158786 27414399 PMC4945021

[49] Tsai HL Chang JW Chen MH Jeng MJ Yang LY Wu KG. Associations between psychiatric disorders and enuresis in Taiwanese children: a national population-based study. Clin Epidemiol. (2020) 12:163–71. 10.2147/CLEP.S230537 32110107 PMC7035896

[50] Gor RA Fuhrer J Schober JM. A retrospective observational study of enuresis, daytime voiding symptoms, and response to medical therapy in children with attention deficit hyperactivity disorder and autism spectrum disorder. J Pediatr Urol. (2010) 8(3):314–7. 10.1016/j.jpurol.2010.10.009 21131234

[51] Kovacevic L Wolfe-Christensen C Rizwan A Lu H Lakshmanan Y. Children with nocturnal enuresis and attention deficit hyperactivity disorder: a separate entity? J Pediatr Urol. (2017) 14(1):47.e1–6. 10.1016/j.jpurol.2017.07.002 28867160

[52] Franco I. Pediatric overactive bladder syndrome. Pathophysiology and management. Pediatr Drugs. (2007) 9(6):379–90. 10.2165/00148581-200709060-00005 18052408

[53] van Leerdam FJ Blankespoor MN van der Heijden AJ Hirasing RA. Alarm treatment is successful in children with day- and night-time wetting. Scand J Urol Nephrol. (2004) 38(3):211–5. 10.1080/00365590410025460 15204373

[54] Hagstroem S Rittig S Kamperis K Djurhuus JC. Timer watch assisted urotherapy in children. A randomised controlled trial. J Urol. (2010) 184(4):1482–8. 10.1016/j.juro.2010.06.024 20727552

[55] Burgers R de Jong TP Visser M Di Lorenzo C Dijkgraaf MG Benninga M. Functional defecation disorders among children with lower urinary tract symptoms. J Urol. (2013) 189(5):1886–91. 10.1016/j.juro.2012.10.064 23123369

[56] Veiga ML Lordêlo P Farias T Barroso C Bonfim J Barroso UJ. Constipation in children with isolated overactive bladder. J Pediatr Urol. (2013) 9:945–9. 10.1016/j.jpurol.2013.01.013 23462384

[57] Burgers R Liem O Canon S Mousa H Benninga MA Di Lorenzo C Effect of rectal distention on lower urinary tract function in children. J Urol. (2010) 184(Suppl 4):1680–5. 10.1016/j.juro.2010.03.120 20728187

[58] Panayi DC Khullar V Digesu GA Spiteri M Hendricken C Fernando R. Rectal distension: the effect on bladder function. Neurourol Urodyn. (2011) 30(3):344–7. 10.1002/nau.20944 21268098

[59] Franco I. The central nervous system and its role in bowel and bladder control. Curr Urol Rep. (2011) 12:153–7. 10.1007/s11934-010-0167-8 21240642

[60] Loening-Baucke V. Prevalence rates for constipation and faecal and urinary incontinence. Arch Dis Child. (2007) 92:486–9. 10.1136/adc.2006.098335 16857698 PMC2066162

[61] Borch L Hagstroem S Bower WF Siggaard Rittig C Rittig S. Bladder and bowel dysfunction and the resolution of urinary incontinence with successful management of bowel symptoms in children. Acta Paediatr. (2013) 102(5):e215–20. 10.1111/apa.12158 23368903

[62] Kim JH Lee JH Jung AY Lee JW. The prevalence and therapeutic effect of constipation in pediatric overactive bladder. Int Neurourol J. (2011) 15(4):206–10. 10.5213/inj.2011.15.4.206 22259734 PMC3256305

[63] De S Teixeira-Pinto A Sewell JR Caldwell PH. Prevalence, patient and consultation characteristics of enuresis in Australian paediatric practice. J Paediatr Child Health. (2018) 54(6):620–4. 10.1111/jpc.13834 29292564

[64] Hsiao YC Wang JH Chang CL Hsieh CJ Chen MC. Association between constipation and childhood nocturnal enuresis in Taiwan: a population-based matched case-control study. BMC Pediatr. (2020) 20(1):35. 10.1186/s12887-020-1939-z 31992241 PMC6986027

[65] Sampaio C Sampaio Sousa A Fraga LGA Veiga ML Bastos Netto JM Barroso U Jr. Constipation and lower urinary tract dysfunction in children and adolescents: a population-based study. Front Pediatr. (2016) 4:101. 10.3389/fped.2016.00101 27752507 PMC5046079

[66] Söderström U Hoelcke M Alenius L Söderling A-C Hjern A. Urinary and faecal incontinence: a population-based study. Acta Paediatr. (2004) 93:386–9. 10.1111/j.1651-2227.2004.tb02966.x 15124844

[67] Naseri M Hiradfar M. Monosymptomatic and non-monosymptomatic nocturnal enuresis: a clinical evaluation. Arch Iran Med. (2012) 15(11):702–6. PMID: 23102248

[68] Sarici H Telli O Ozgur BC Demirbas A Ozgur S Karagoz MA. Prevalence of nocturnal enuresis and its influence on quality of life in school-aged children. J Pediatr Urol. (2016) 12(3):159.e1–6. 10.1016/j.jpurol.2015.11.011 26778419

[69] Rodríguez-Ruiz M Mendez-Gallart R García Mérida M Somoza-Argibay I. Influence of constipation on enuresis. An Pediatr (Engl Ed). (2021) 95(2):108–15. 10.1016/j.anpedi.2020.06.016 34373073

[70] Kajiwara M Inoue K Kato M Usui A Kurihara M Usui T. Nocturnal enuresis and overactive bladder in children: an epidemiological study. Int J Urol. (2006) 13(1):36–41. 10.1111/j.1442-2042.2006.01217.x 16448430

[71] Borgström M Bergsten A Tunebjer M Hedin Skogman B Nevéus T. Fecal disimpaction in children with enuresis and constipation does not make them dry at night. J Pediatr Urol. (2022) [online ahead of print]. 10.1016/j.jpurol.2022.05.008 35718673

[72] Burgers RE Mugie SM Chase J Cooper CS von Gontard A Rittig CS Management of functional constipation in children with lower urinary tract symptoms; Report from the Standardization Committee of the International Children’s Continence Society. J Urol. (2013) 190(1):29–36. 10.1016/j.juro.2013.01.001 23313210

[73] Nieuwhof-Leppink AJ Hussong J Chase J Larsson J Renson C Hoebeke P Definitions, indications and practice of urotherapy in children and adolescents:—a standardization document of the International Children’s Continence Society (ICCS). J Pediatr Urol. (2021) 17(2):172–81. 10.1016/j.jpurol.2020.11.006 33478902

[74] Pennesi M Pitter M Bordugo A Minisini S Peratoner L. Behavioral therapy for primary nocturnal enuresis. J Urol. (2004) 171(1):408–10. 10.1097/01.ju.0000097497.75022.e8 14665944

[75] Baeyens D Lierman A Roeyers H Hoebeke P Vande Walle J. Adherence in children with nocturnal enuresis. J Pediatr Urol. (2009) 5(2):105–9. 10.1016/j.jpurol.2008.10.002 18996052

[76] Kurt O Yazici CM Paketci C. Nocturnal enuresis with spina bifida occulta: does it interfere behavioral management success? Int Urol Nephrol. (2015) 47:1485–91. 10.1007/s11255-015-1047-4 26149636

[77] Tkaczyk M Maternik M Krakowska A Wosiak A Miklaszewska M Zachwieja K Evaluation of the effect of 3-month bladder basic advice in children with monosymptomatic nocturnal enuresis. J Ped Urol. (2017) 13(6):615.e1–6. 10.1016/j.jpurol.2017.03.039 28634090

[78] Cederblad M Engvall G Sarkadi A Nevéus T. No effect of basic bladder advice in enuresis—a randomised controlled trial. J Ped Urol. (2015) 11(3):153e1–5. 10.1016/j.jpurol.2015.03.004 25975733

[79] Borgström M Bergsten A Tunebjer M Hedin Skogman B Nevéus T. Daytime urotherapy in nocturnal enuresis—a randomised, controlled trial. Arch Dis Child. (2022) 107(6):570–4. 10.1136/archdischild-2021-323488 35074830 PMC9125372

